# The RADAR coverage tool: developing a toolkit for rigorous household surveys for reproductive, maternal, newborn, and child health & nutrition indicators

**DOI:** 10.1080/16549716.2021.2006419

**Published:** 2022-09-13

**Authors:** Melinda K Munos, Abdoulaye Maïga, Talata Sawadogo-Lewis, Emily Wilson, Onome Ako, Serafina Mkuwa, Frida Ngalesoni, Jennifer L. Brenner, Dismas Matovelo, Idrissa Ouili, Abdramane Soura, Moussa Bougma, Ashley Sheffel, Amy J Hobbs, Neff Walker

**Affiliations:** a Department of International Health, Johns Hopkins Bloomberg School of Public Health, Baltimore, Maryland, USA; b Amref Health Africa, Toronto, Canada; c Amref Health Africa in Tanzania, Dar Es Salaam, Tanzania; d Departments of Pediatrics and Community Health Sciences, Cumming School of Medicine, University of Calgary, Calgary, Canada; e Catholic University of Health and Allied Sciences – Bugando, Mwanza, Tanzania; f Institut Supérieur Des Sciences de La Population, Université Joseph Ki-Zerbo, Ouagadougou, Burkina Faso

**Keywords:** Household survey, maternal health, newborn health, child health, nutrition

## Abstract

Population-based intervention coverage data are used to inform the design of projects, programs, and policies and to evaluate their impact. In low- and middle-income countries (LMICs), household surveys are the primary source of coverage data. Many coverage surveys are implemented by organizations with limited experience or resources in population-based data collection. We developed a streamlined survey and set of supporting materials to facilitate rigorous survey design and implementation. The RADAR coverage survey tool aimed to 1) rigorously measure priority reproductive, maternal, newborn, child health & nutrition coverage indicators, and allow for equity and gender analyses; 2) use standard, valid questions, to the extent possible; 3) be as light as possible; 4) be flexible to address users’ needs; and 5) be compatible with the Lives Saved Tool for analysis of program impact. Early interactions with stakeholders also highlighted survey planning, implementation, and analysis as challenging areas. We therefore developed a suite of resources to support implementers in these areas. The toolkit was piloted by implementers in Tanzania and in Burkina Faso. Although the toolkit was successfully implemented in these settings and facilitated survey planning and implementation, we found that implementers must still have access to sufficient resources, time, and technical expertise in order to use the tool appropriately. This potentially limits the use of the tool to situations where high-quality surveys or evaluations have been prioritized and adequately resourced.

## Background

Effective interventions exist to prevent or reduce most major causes of maternal, neonatal, and child morbidity and mortality [1–3], yet the burden of morbidity and mortality remain high, particularly in low- and middle-income countries (LMICs). Intervention coverage, or the proportion of individuals in need of a service or intervention who receive that service or intervention, provides information on the extent to which interventions are reaching populations in need. Intervention coverage can also be stratified by socio-demographic covariates, such as sex, wealth, education, urbanicity, or geography to assess inequities and identify underserved populations. Intervention coverage is used as an outcome in many program evaluations, since increases in population coverage of effective interventions are expected to lead to improvements in health status, and it is often faster and less expensive to capture changes in intervention coverage than it is to measure changes in health status.

There are two main sources of intervention coverage data: population-based surveys, and data reported routinely by health facilities. The Demographic and Health Surveys (DHS) [4] and the Multiple Indicator Cluster Surveys (MICS) [5] are nationally representative household surveys which provide population and health data that is useful for obtaining coverage estimates and evaluating programs. However, they are resource- and time-intensive and require a high level of technical skill to implement, and are therefore conducted infrequently. While many countries implement DHS and/or MICS or equivalent surveys every 3–5 years, some countries may go 10 years or more without a national health and demographic survey.

Obtaining accurate and meaningful measures of coverage outside of a DHS or MICS can be challenging. Although it is preferable to use existing sources of coverage data (DHS, MICS, routine facility data) to inform program design and assess program impact, this is not always feasible. Data may not exist for the time period(s), geographical area(s), population(s), and/or indicators of interest, and/or the sample size may be insufficient to support planned analyses. In these cases, program implementers and evaluators must consider whether to conduct their own household survey(s), weighing the considerable time and resource requirements for these surveys against the additional information that the surveys would provide.

The DHS and MICS questionnaires, which measure a wide range of intervention coverage and health indicators, are publicly available. DHS and MICS also share supporting materials, including interviewer manuals, guides to analyzing MICS and DHS data, and tabulation plans [4,5]. Because these surveys have been implemented for decades and include questions that have been piloted, and in some cases validated [6–15], in many settings, the questionnaires and associated materials are an important resource for organizations seeking to conduct their own household surveys. However, the breadth and depth of these questionnaires mean that they are technically complex, time-consuming, and expensive to implement. This can make them challenging to adapt for organizations with limited household survey experience and budgets. There is a need for a less complex tool focused on RMNCAH&N that can be easily adapted and implemented by a wide range of organizations, while maintaining methodological rigor.

The Real Accountability: Data and Analysis for Results (RADAR) project, supported by Global Affairs Canada (GAC), aimed to improve the evidence base for effective programming for women and children by developing and testing tools to improve RMNCAH&N program accountability. The RADAR coverage survey tool aims to answer the question ‘Do women and children who need interventions actually receive them?’ within the overall RADAR evaluation framework. This paper describes the tool, its development, and pilot implementation, and discusses the challenges identified during tool development and use. Because the tool intentionally measures standard indicators (except for the gender indicators, which are discussed in a separate publication), this paper focuses primarily on the development of the tool and on implementation experiences.

## Tool development

### Overview

The RADAR coverage tool was developed by a team of epidemiologists and demographers with experience in coverage survey design, implementation, and analysis in LMICs. Prior to starting development of the RADAR coverage tool, we established five principles to guide tool development. Namely, the tool should: 1) Measure core RMNCAH&N coverage indicators; 2) use standard, validated questions, to the extent possible; 3) be as ‘light’ (short) as possible; 4) remain flexible in order to address programming needs; and 5) be compatible with the Lives Saved Tool (LiST) – a model that allows users to estimate the impact of coverage change on health outcomes [16].

To inform tool development, we held a series of workshops with stakeholders, and conducted three pilot implementations of the tool – one led by the RADAR team, and two by other implementing organizations with JHU support. In 2015, we held a workshop with senior scientists from academic institutions with expertise in household survey research to examine issues in creating a light, easily adaptable and implementable household survey. These issues included re-examining the minimum data needed for a coverage survey, considering how to simplify the sampling design, and streamlining survey management, data management, and analysis. Based on these discussions, we decided that promoting high-quality data was a priority, leading us to recommend the use of rigorous probability sampling and a household roster to identify eligible respondents, even though these would increase the time and budget required. To streamline the tool, then, we focused on including only those questions necessary to measure the priority indicators of the funder and partners.

By mid-2016, early versions of the questionnaires and some of the accompanying tools (manuals, excel-based sample size calculators) were available. In 2016–17, we shared these tools with with organizations who were conducting household surveys for their GAC-funded initiatives – primarily Canadian non-governmental organizations (NGOs) and academic institutions – conducted workshops and meetings with these organizations to understand users’ needs and constraints, and incorporated the feedback into the tool development and revisions. We also consulted with a gender expert who developed a module of gender-sensitive indicators and questions based on Morgan et al.’s framework [17] and gender questions in the DHS and MICS, to allow for gender analyses with the survey data.

### Indicator selection and questionnaire development

Although one of our goals was to produce a ‘light’, or short, coverage survey, we found that many users wanted to include additional indicators. There was an ongoing tension between the need to keep the survey light, and the need to provide a tool that collected the indicators needed by its users and by GAC, which required a certain number of questions. Although we started with a list of 18 priority MNCH&N indicators prioritized by GAC (Web Appendix 1), many implementers indicated a desire to measure additional indicators that were important to their initiatives. We added additional 31 RMNCAH&N indicators, focusing on those (1) requested by users, (2) that measure intervention coverage, and (3) for which standard, tested, easy to implement questions existed. In addition, as sexual and reproductive health and rights (SRHR) and gender became an increasingly important area of focus for GAC, we added 12 SRHR indicators and 8 categories of gender indicators. The complete list of indicators is available in on the RADAR website (https://www.radar-project.org/coverage-survey). The development and analysis of the gender questions is described elsewhere [18]. Examples of indicators that stakeholders requested but that we ultimately decided not to add included: under-five and maternal mortality (not coverage; too burdensome to collect); knowledge and attitudes (not coverage); intrapartum interventions (evidence of invalidity); and women’s experience of gender-based violence (requires very strong capacity, confidentiality protections, and adherence to ethical norms, which we could not guarantee).

In order to ensure compatibility with LiST, we harmonized indicator definitions with the interventions included in LiST, to the extent possible. However, we note that LiST includes many interventions that are difficult or impossible to measure in a household survey (for example, care delivered to the mother during the intrapartum period); LiST often uses health facility surveys to estimate these indicators. In addition, some LiST interventions are possible to measure but are not yet widely implemented in most LMICs (for example, maternal micronutrient supplements in pregnancy, or preventive zinc supplementation in children). In the interest of maintaining a streamlined questionnaire, we did not include these interventions.

**Figure 1.**
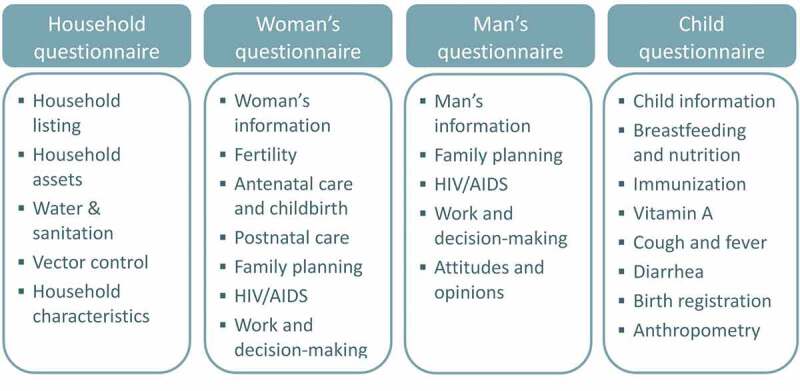
RADAR Coverage Survey Questionnaires and Modules.

We adopted a modular questionnaire structure, similar to the MICS questionnaires [5], to facilitate adaptation by partners. We started with the list of indicators, identified the standard questions necessary to measure those indicators, and constructed the survey questionnaires from those questions. We also included key stratifiers (sex, age, education, wealth) and a household roster to identify household members eligible for the individual questionnaires (Figure 1). Questions and response options requiring country- or program- level adaptation were flagged in the questionnaires. Rather than using a full birth history to identify births in the past two years, the RADAR tool asks the respondent only about her most recent live birth in the previous two years, similar to earlier rounds of the MICS. This greatly simplifies both data collection and analysis.

During the pilot implementations, the questionnaires were translated into French and Swahili. DHS and MICS questionnaires in French and Swahili were used as a basis for the translation to ensure that the tool would produce comparable data to these surveys. The questionnaires were reviewed by team members or consultants who were fluent in French and Swahili and had substantial experience conducting coverage surveys to verify the translation. Translation issues identified during the pilot implementations were corrected in the questionnaires. The final questionnaires are available on the RADAR website.

### Pilot implementations of the tool

The tool was piloted with two implementers in Tanzania. In August-October 2016, the first pilot was conducted with the Mama na Mtoto initiative involving the Catholic University of Health and Allied Sciences – Bugando (CUHAS), the University of Calgary, and Mbarara University of Science and Technology using an early version of the RADAR coverage tool. This pilot was a 2000 household survey in Misungwi district, Mwanza region, Tanzania with supplementary technical support from the JHU RADAR coverage team. The second pilot was conducted in November 2017-March 2018 by Amref Health Africa’s Canada and Tanzania offices. Amref implemented the coverage tool in a 2000 household survey in Simiyu region, Tanzania, with technical and financial support from RADAR (which is funded by Global Affairs Canada). Both Tanzania surveys served as the baseline for internal, pre-post evaluations of the implementers’ program, and implementers also used the results to inform program design. The sample size for each survey was based on the objectives, available resources, and needs of the evaluations. During each pilot of the tool, an excel workbook (template available as Web Appendix 2) was used to document issues encountered and resolutions. Each tool pilot survey was followed by a round of revisions to the tool based on the documented issues.

After the Tanzania RADAR implementation experiences, a near-final version of the tool was implemented in Burkina Faso in November 2019 – March 2020 in collaboration with the Institut Supérieur des Sciences de la Population (ISSP), as part of a study testing a novel household sampling approach. This was a three-arm study that sampled 3000 households per arm, for a total of 9000 households.

In addition to these pilot implementations, which took place with technical support from the RADAR team, we made beta versions of the toolkit components available to other organizations receiving funding from GAC. Several of these organizations indicated that they have used components of the toolkit (e.g. sample size calculator, questionnaire, Open Data Kit (ODK) questionnaire) without technical support from RADAR. However, as the toolkit was widely distributed and is now available on a website, it has been difficult to track or quantify broader use of the tool.

### Development of the survey toolkit

Interactions with implementing organizations at workshops and during tool pilots highlighted the challenges that many organizations, particularly those whose focus is on program implementation, experience in planning and managing surveys, and in analyzing and interpreting survey data. We therefore prioritized the development of supporting materials to facilitate survey planning and implementation.

## Description of tool

The RADAR coverage survey includes four questionnaires: a household questionnaire, woman’s questionnaire, man’s questionnaire, and an under-five questionnaire (Figure 1). The RADAR coverage toolkit also includes materials designed to support the coverage survey process, including survey design and planning, sampling and mapping, training and data collection, and data management and analysis (Table 1). The materials are online and publicly available on the RADAR project website (https://www.radar-project.org/coverage-survey). To maximize the utility of the toolkit, we designed the materials to be useable with questionnaires other than the RADAR questionnaire, to the extent possible. For example, the survey planning, sampling, mapping, and cross-cutting guidance materials can all be used independently of the RADAR questionnaires. The manuals and data management and analysis materials are necessarily linked to the RADAR questionnaires, but could be adapted for use with similar questionnaires. The questionnaires are primarily designed for computer-assisted personal interviewing (CAPI), but the Word version of the questionnaire can be used for interviewer training and for paper-based data-collection. The CAPI tools are usable on any Android device, including tablets and mobile phones.

**Table 1.** RADAR toolkit content.

Survey phase	Supporting materials	Description
Design/Planning	Budget and timeline templates, and linked supplies list	Tools to help guide survey planning and logistics. Results from the supplies list can be copied/pasted directly into the budget tool.
	Sample size calculator	Online tool to help users calculate the sample size needed to measure their indicators of interest in their target population.
	Indicator sheets	Detailed definitions of all indicators that can be calculated from the RADAR coverage survey tool, including numerator, denominator, questions required for each indicator, and notes relevant for indicator calculation.
Sampling/Mapping	Cluster sampling tool	Desktop and excel-based application to sample clusters with probability proportional to population size.
	Mapping manuals, presentations, forms, and Open Data Kit (ODK) form	Guidance documents and materials to be used during training and implementation of household mapping/enumeration.
	Household sampling tool	Desktop and excel-based application to sample households using systematic random sampling.
	Deployment plan template	Template to guide the development of a deployment plan for mapping fieldwork.
Training and data collection	Interviewer and supervisor manuals and forms	Detailed manuals explaining the questionnaire question-by-question and providing guidance on how to conduct fieldwork and the roles and skills necessary for fieldwork for interviewers and supervisors. Manual sections that must be modified to users’ specific needs are highlighted in color, and they are designed to be used during training and during data collection. The manuals include sections on consent and confidentiality, as well as data quality assurance.
	Training agenda, presentations, and learning assessments	Tools for interviewer training, intended to be paired with the manuals and questionnaires for a comprehensive training. Sections that must be modified for the specific survey are highlighted.
	Word questionnaires	Questionnaires in Word format that can be used during interviewer training and/or for paper-based data collection.
	ODK questionnaires and manual	Open Data Kit (ODK) is an open-source computer-assisted personal interviewing (CAPI) platform. The RADAR ODK questionnaires contain the complete RADAR coverage survey, in ODK Excel format, for data capture on Android tablets. The RADAR ODK manual supplements open-source documentation on ODK to aid users who are implementing ODK surveys.
Data management & analysis	Data quality assessment indicators	Table of indicators (and corresponding R code) that can be used to assess the quality of incoming data and flag potential issues for follow-up in the field.
	Data processing scripts	Series of STATA .do files (code) that supports data management including cleaning variable names, final data cleaning, data preparation, and data archiving.
	Data analysis and tabulation scripts	Series of STATA .do files (code) that supports indicator generation and tabulation accounting for a complex survey design (requires adaptation by users to account for their survey design).
	Gender analysis guide	Describes rationale for including gender analysis in coverage surveys, and offers templates and quantitative examples of gender analysis from RADAR implementation.
Cross-cutting guidance	Implementation guide	Comprehensive step-by-step guide for entire coverage survey RADAR survey process from planning to data analysis.
	Coverage survey course	Online course introducing users to coverage surveys, presenting RADAR coverage survey tools, and guiding them through the steps to implement a coverage survey.

## Tool implementation experiences and challenges

The data from the pilot survey implementations are, or will be, available in separate reports [19,20]. Here we describe the experiences and challenges encountered during these pilots, as well as key feedback from implementers and users with whom we engaged during workshops and other meetings.

### Implementers face a variety of challenges when implementing high quality coverage surveys

We found that many implementers (which included non-governmental organizations, academic institutions, membership-based organizations, and other development partners who had received funding from GAC to implement RMNCAH&N initiatives) had limited experience conducting household surveys at the beginning of the project, as their main function was program design and implementation. The exception to this was academic institutions with extensive evaluation experience. As a result, some implementers, and particularly those that were not research institutions, relied on consultants for survey design, implementation, and analysis, or completed these tasks in-house but with some difficulty. Some implementers also highlighted that data collection activities diverted staff and resources away from program activities, potentially delaying program implementation.

Difficulties experienced during one or more pilot implementations or reported by other users included estimating the sample size, securing sufficient budget for the survey, logistical planning for the survey, adapting survey questionnaires and developing a computer-assisted personal interviewing (CAPI) tool, sampling clusters and households using probability sampling, training mapping and survey teams, ensuring data quality during data collection, and analyzing hierarchical data accounting for the complex survey design. The contents of the coverage survey toolkit were developed in response to these observations to try to alleviate these challenges. During workshops, several organizations also noted that when they hired consultants to conduct household surveys, it was challenging to evaluate their quality of the work and the data produced.

### Sample sizes and budgets were sometimes too small to measure indicators of interest

In several cases, there was a lack of alignment between the indicators that the implementing organizations sought to measure (or were asked to measure by the funder) and the planned sample size and budget for the survey. In one case, the funder asked that the survey measure coverage indicators for adolescents (for example, skilled birth attendant among women 15–19 years). We estimated that a sample size of 7000 households would be needed to measure skilled birth attendant among women 15–19 years with precision of 5 percentage points, or 2000 households with precision of 10 percentage points. However, the implementer’s budget was less than half what would be needed for a 2000 household survey. Similarly, another organization hoped to measure maternal and child mortality and nutrition impact using a 2000 household survey, although this sample size was insufficient to estimate maternal or child mortality with acceptable precision.

A sample size or budget that is smaller than what is needed for the targeted indicators can lead to wasted resources and data, if the sample is too small to draw useful inferences. This may mean that actual changes in coverage are not detected (Type II error), or that very imprecise point estimates are used to inform program design. The RADAR sample size calculator was developed to help organizations easily estimate sample size requirements for their indicators of interest and communicate these to funders and other stakeholders. This calculator was initially in Excel, but based on feedback and observations about difficulties using the form, we converted it to a web-based application available at http://www.radarsamplesizecalculator.org/.

### Conducting probability sampling was a key challenge that could not be fully resolved

Conducting high-quality probability sampling was one of the most common challenges mentioned by potential users. We recommended standard probability sampling approaches, including mapping and enumerating households within sampled clusters to establish a sampling frame of households. During the pilot implementations and workshops, organizations identified a number of barriers to conducting probability sampling, including cost; time, particularly given grant constraints (e.g. 6 months to plan and carry out the baseline survey); and lack of experience with this method. Although we developed manuals, mapping tools, and data collection tools, this was the area that required the most technical assistance from the RADAR team during pilots; the RADAR tools were not able to address all the constraints to conducting probability sampling.

Compounding these challenges, the RADAR team recommended using experienced mapping personnel to ease the burden of work, shorten time in the field, and ensure good data quality. However, recruiting mappers who resided in or near the survey area, knew the local customs and language, and had experience in mapping was a challenge in several of the pilot implementations. The training for both Tanzanian pilot surveys was conducted in Mwanza district. It was difficult to find experienced mappers in this area since the National Bureau of Statistics (NBS), which conducts much of the mapping in Tanzania, is located elsewhere, and there were no recent household surveys based in Mwanza. For the first pilot, individuals with no mapping experience were recruited from Mwanza and surrounding areas. This required significant training for both the mappers and for some of the survey team. Conversely, in another pilot, the implementer prioritized previous experience for their recruitment of mappers. They worked with Tanzania’s NBS to identify and sample clusters in the survey area and to recruit mappers with previous experience. Since most of these mappers were located elsewhere in the country, they had to travel to Mwanza for the training. Establishing this arrangement with NBS and transporting and housing these mappers in Mwanza and the survey area added to the cost and time for the survey. The collaboration with NBS (and, in Burkina Faso, with the Institut National de la Statistique et de la Démographie) had the positive effect of promoting ownership of the survey by the countries statistical bureaus.

### Designing easily adaptable data management systems was a challenge

Another commonly mentioned challenge was data management and analysis. All three pilot surveys implemented the RADAR Coverage Tool using ODK on tablets. The data managers for the surveys were part of the implementing organizations or, in one case, a consultant. The amount of technical assistance for data management provided by the JHU RADAR team varied by survey. The data management tasks most frequently requiring support from RADAR included: providing and adapting the ODK questionnaire, setting up the server, and creating a data quality dashboard. Navigating tablets, field-testing the ODK questionnaire, and reviewing incoming data using the data quality dashboard could sometimes be done by the data manager or other implementer staff.

A generic ODK questionnaire is available for the RADAR tool, along with a manual to supplement the ODK documentation, but it requires some adaptation (editing response options, dropping or adding modules or questionnaires). We chose ODK because it is free, open access, and relatively straightforward to learn. However, learning to make adaptations to an ODK questionnaire and troubleshoot any issues still requires dedicated time, which is not always available to staff who have many other responsibilities.

Although we developed and used data quality dashboards to monitor incoming data in each of the pilot implementations, we were not able to develop a version that was easily adaptable by implementers. We also found that it was challenging to ensure that supervisors and central office staff were reviewing and critically assessing incoming data for potential quality issues, although we revised training materials after each pilot implementation to try to address this shortcoming. These tasks required substantial training and experience in interpreting and using survey data quality indicators, as well as sufficient time for survey supervisors and managers to review data and communicate back to teams, and this time was often in short supply during survey implementation.

## Discussion and conclusion

Coverage surveys are an important tool for effectiveness evaluations of health programs, providing information on who is and is not receiving interventions, and trends over time. However, these surveys must be implemented with good quality in order to provide accurate and meaningful information. This includes appropriate selection of indicators for the program evaluation, correct sample size calculations, an appropriate sampling design, well-designed questionnaires and data management systems, high-quality training and supervision, and data analysis that appropriately accounts for the sampling design. The RADAR coverage tool and toolkit aims to provide users with tools to support the design, planning, implementation, and analysis of their surveys, as well as a structured questionnaire that reflects best practices and information about question validity.

The RADAR coverage toolkit has a number of strengths. It focuses on questions required to measure standard coverage indicators for program evaluation and equity analyses for RMNCAH&N, and organizes these in a modular format to make adaptation easier. This results in a survey measuring approximately 70 indicators. While a direct comparison to MICS and DHS is difficult because each survey differs in scope and purpose, and DHS does not have a publicly available list of all indicators, the MICS includes over 175 indicators, and the DHS questionnaires are substantially larger than the MICS. Similar to earlier versions of the MICS, the RADAR survey also streamlines collection of data on maternal and newborn indicators by asking only about the most recent live birth rather than requiring a full birth history. The tool includes a gender module and integrates questions required for gender-based analysis. It also distinguishes between those modules which are essential versus those which are optional for organizations with limited resources and household survey experience. Most importantly, it provides a comprehensive set of materials to support organizations conducting coverage surveys – including a sample size calculator, sampling and mapping tools, training materials, CAPI tools, analytical files, and an online course.

The RADAR coverage toolkit also has limitations. A major difficulty identified by many implementers was probability sampling, which requires mapping clusters and enumerating all households in those clusters. We explored several alternatives and tested and costed a GIS/satellite image-based method at scale; one of the pilot survey implementers also tried a modified sampling approach that sampled ‘wedges’ of clusters; these will be published separately and the GIS sampling materials will be provided. We did not test simultaneous mapping and data collection [21], which somewhat reduces overall time in the field, but which requires very strong supervision and organization of data collection. Ultimately, we were unable to significantly reduce the time, resources, and technical expertise required to conduct probability sampling for in-person surveys. We urge funders to support high quality sampling by ensuring that they are asking about sampling methods, adequately resourcing surveys, and allowing implementers enough time to conduct the survey.

We also were not able to develop an easily adaptable data quality dashboard for incoming survey data, so we have not included this in our toolkit. While we did develop a dashboard, it needed to be recoded for each implementation to account for the adaptations to the survey (questionnaires/modules included, changes to the response codes, etc.). We have, however, made available our list of data quality indicators and the corresponding R script for calculating these indicators.

The RADAR coverage tool is intended to be used in situations where there are no existing and appropriate coverage data that can be used. Although we have tried to simplify and streamline the survey, because it is an in-person survey, it is inherently expensive, logistically complicated, and time-consuming to implement, and therefore not suitable for all evaluations. The RADAR coverage tool is also not a guarantee of a high-quality survey or evaluation. Good surveys still require sufficient time (6–9 months including planning and analysis), budget, and well-trained study teams. High-quality evaluations are not limited to baseline and endline surveys and require measurement of intermediate variables and designs that attempt to account for the counterfactual (what would have happened had the program not been implemented) [22]. We cannot overstate the complexity of conducting a high-quality household survey, and we emphasize that the decision to conduct such a survey (or to require organizations receiving funding to conduct one) must consider the organization’s capacity and resources, the opportunity costs and added value of the survey, and the plans for using the survey data to improve our understanding of program effectiveness and/or population health.

On an encouraging note, the end of project data collection process for the Mama na Mtoto initiative repeated use of the RADAR tool and used the toolkit materials to support its implementation. This was conducted by coalition partners without support from RADAR but with support from a consultant who had previously been part of the RADAR team; the study ran smoothly, used an all-new team of data collectors and mappers, and involved in-country experts exclusively during training and supervision. Data collection was completed in over 2000 households in four weeks.

The development of the RADAR toolkit has highlighted important research needs of population-based surveys in LMICs. Mobile phone surveys have the potential to substantially reduce the costs and time required for household coverage surveys [23], and interest in these surveys has increased dramatically as the SARS-CoV-2 pandemic has impeded in-person data collection. However, more evidence is needed to understand what kinds of settings these surveys can perform well in, which respondents are reached, which methods can improve the representativeness of the sample, and which kinds of questions respondents can (accurately) respond to via mobile phone. It is likely that in-person household surveys will continue to be needed in some countries for some time; more research and innovative thinking is needed around less burdensome sampling methods. Finally, it is important to understand which indicators are best measured in a household survey as opposed to alternative approaches, including the use of routine health information systems and electronic health records data, and to continue to improve the availability and quality of these data sources.

## Research in context

Household surveys are used to improve and evaluate programs, but many organizations face challenges conducting these surveys. The RADAR coverage survey tool aims to provide streamlined questionnaires to collect key health coverage indicators, and a toolkit to facilitate survey planning, implementation, and analysis. The RADAR tool was used by organizations with varying degrees of technical assistance. Household survey toolkits can facilitate household survey implementation, but technical capacity and sufficient budget and time are still needed.
